# Diagnosis and control of anthelmintic-resistant *Parascaris equorum*

**DOI:** 10.1186/1756-3305-2-S2-S8

**Published:** 2009-09-25

**Authors:** Craig R Reinemeyer

**Affiliations:** 1 East Tennessee Clinical Research, Inc., 80 Copper Ridge Farm Road, Rockwood, TN 37854 USA

## Abstract

Since 2002, macrocyclic lactone resistance has been reported in populations of *Parascaris equorum *from several countries. It is apparent that macrocyclic lactone resistance developed in response to exclusive and/or excessively frequent use of ivermectin or moxidectin in foals during the first year of life. The development of anthelmintic resistance was virtually inevitable, given certain biological features of *Parascaris *and unique pharmacologic characteristics of the macrocyclic lactones. Practitioners can utilize the Fecal Egg Count Reduction Test to detect anthelmintic resistance in *Parascaris*, and the same technique can be applied regularly to confirm the continued efficacy of those drugs currently in use. In the face of macrocyclic lactone resistance, piperazine or anthelmintics of the benzimidazole or pyrimidine classes can be used to control ascarid infections, but *Parascaris *populations that are concurrently resistant to macrocyclic lactones and pyrimidine drugs have been reported recently from Texas and Kentucky. Compared to traditional practices, future recommendations for ascarid control should feature: 1) use of only those anthelmintics known to be effective against indigenous populations, 2) initiation of anthelmintic treatment no earlier than 60 days of age, and 3) repetition of treatments at the longest intervals which prevent serious environmental contamination with *Parascaris *eggs. In the interest of decreasing selection pressure for anthelmintic resistance, horse owners and veterinarians must become more tolerant of the passage of modest numbers of ascarid eggs by some foals. Anthelmintic resistance is only one of several potential responses to genetic selection. Although still only theoretical, changes in the immunogenicity of ascarid isolates or reduction of their prepatent or egg reappearance periods could pose far greater challenges to effective control than resistance to a single class of anthelmintics.

## 

Anthelmintic resistance in parasites of horses was first reported approximately five decades ago when various researchers noted that phenothiazine treatment failed to reduce strongylid egg counts [1-3]. Anthelmintic resistance in cyathostomin (small strongyle) nematodes has since expanded to encompass nearly universal insusceptibility to benzimidazoles [4,5], resistance to pyrantel salts by nearly 50% of populations in the U.S. [4-6], and occasional resistance to piperazine [7]. Lyons *et al. *[8] recently reported shortened egg reappearance periods and survival of fourth-stage larval cyathostomins following treatment with macrocyclic lactone anthelmintics. These phenomena are considered to be companions and precursors of clinical anthelmintic resistance.

Yet, most concerns that parasitologists and equine practitioners harbored about anthelmintic-resistant cyathostomins were mitigated by the fact that small strongyles are generally not serious pathogens in well-managed horses. Concern was amplified into alarm, however, by the first published reports of anthelmintic resistance in *Parascaris equorum *[9,10]. *P. equorum *is the most pathogenic parasite of juvenile equids, and can cause poor growth, ill-thrift, weight loss, colic, and death subsequent to intestinal impaction or perforation.

Although the parasitology community was taken aback by the development of macrocyclic lactone (ML) resistance in a non-strongylid nematode, an honest assessment of historical management practices for equine ascarids, with due application of resistance selection theory, should have predicted this circumstance. In retrospect, perhaps the most surprising element about the development of macrocyclic lactone resistance in equine ascarids is that it did not arise until nearly 20 years after the first approval of ivermectin for horses.

Horse owners and equine practitioners are now aware of anthelmintic-resistance in ascarids, and have numerous practical questions regarding its detection, management, and prevention. The objectives of this paper are to review the current status of anthelmintic resistance in populations of *P. equorum*, to discuss the biological and management factors which promoted its development, and to offer practical methods of detection, chemical control, and prevention for breeding stables.

## Life cycle

*P. equorum *(ascarid; roundworm) is a common nematode parasite which occurs in the small intestine of immature horses world-wide. Adult female ascarids lay eggs in the small intestine, and these eggs pass into the environment within the feces of the host. The infective stage is a larvated egg (containing a second stage larva [L]_3_); development requires approximately 10 days at temperatures of 25°C to 35°C [11]. Larvated eggs survive in the environment for up to five or 10 years, and infection is acquired through inadvertent ingestion of eggs. Larvae emerge from eggs within the alimentary tract of a horse, and migrate through the liver and lungs before returning to the small intestine approximately one month later as fourth stage larvae (L_4_). Ascarids mature progressively in the small intestine and achieve patency about 75 to 80 days after infection [11].

*P. equorum *is one of the rare nematodes which induces absolute acquired immunity. Most horses become immune during the first year of life, so patent ascarid infections are rarely diagnosed in horses over two years of age.

## Anthelmintic resistance

Failures of macrocyclic lactone treatment to decrease *Parascaris *fecal egg counts were first reported in the Netherlands [9] and Canada [10]. Subsequently, macrocyclic lactone-resistant (ML-R) populations of *P. equorum *have been detected in numerous countries, including the United States [12,13], Denmark [14], Germany [15], Brazil [16], and Italy [17]. A comprehensive survey of the distribution of ML-R *Parascaris *populations has not been conducted, but anecdotal reports abound in North America.

The initial clinical evidence of macrocyclic lactone resistance (ML-R) consisted of failures of ivermectin (IVM) or moxidectin (MOX) to decrease ascarid egg counts after treatment. To characterize this phenomenon more thoroughly, an efficacy study was conducted in 2005 with 11 foals that had been raised helminth-free. These foals were inoculated orally at 6 weeks to 3 months of age with ~500 larvated eggs of a Canadian isolate of *P. equorum *that was purportedly resistant to macrocyclic lactone anthelmintics [18]. Six foals were treated orally with ivermectin paste (200 μg/kg), and the remaining five served as untreated controls. Ivermectin treatment did not result in significant Fecal Egg Count Reduction (FECR), and worm numbers at necropsy were decreased by only 22%. This study unequivocally confirmed ivermectin resistance in *P. equorum*, and a subsequent study wherein alternating treatments of ivermectin and moxidectin failed to reduce egg counts demonstrated that such resistance involved the entire macrocyclic lactone class [19].

## Inherent factors contributing to resistance

All currently marketed equine anthelmintics are considered to be "broad spectrum", meaning they have good efficacy (>90%) against four groups of target parasites: large strongyles, cyathostomins, ascarids, and pinworms. Broad spectrum anthelmintics are not uniformly effective against all parasitic targets; invariably, one parasite always requires a higher dosage than the others to achieve efficacy [20]. These hardest-to-kill species are known as dose-limiting parasites (DLPs), and *P. equorum *is the DLP for most equine anthelmintics.

The clearest example of *Parascaris *as a DLP is seen with fenbendazole (FBZ). In horses, the 5 mg/kg dosage of FBZ is effective against large strongyles, susceptible cyathostomins, and pinworms, but the recommended dosage for removal of *Parascaris *is 10 mg FBZ/kg body weight.

Because the magnitude of difference between an effective dosage and the label dosage is much less for DLPs than for other intended targets, dose-limiting parasites have a lower threshold for the development of resistance.

## Pharmacologic factors selecting for resistance

Macrocyclic lactones are the most persistent anthelmintics used in horses, and effective drug levels may persist in the plasma for days to weeks after a single treatment. Drug concentrations inevitably decline, however, and parasites that are newly acquired during this phase may be exposed to subtherapeutic concentrations as a consequence. Low drug concentrations during the decay phase of persistent products can select for anthelmintic resistance (so-called "tail selection") [21].

In contrast, anthelmintics of the pyrimidine and benzimidazole classes are non-persistent. Resistance of *P. equorum *to pyrantel pamoate has been reported recently in herds from Texas and Kentucky [12,13]. Pyrantel pamoate resistance was possibly pre-selected by daily use of pyrantel tartrate in some herds for prevention of ascarid and strongyle infections. Pyrantel-resistant ascarids have not been reported outside of North America, which is the exclusive marketing range of pyrantel tartrate for prophylactic use in horses [5]. Ascarid resistance to benzimidazoles has not been reported in North America, perhaps because use of this class has been limited to non-persistent, therapeutic applications.

## Control practices which select for resistance

Anthelmintics are used excessively by many breeding farms, where it is a common practice to administer ivermectin for treatment of suspected *Strongyloides *infection when foals are less than one month of age. Thereafter, frequent anthelmintic rotation is implemented, and juvenile horses are often dewormed at monthly intervals until their first birthday. Many farms use macrocyclic lactones at least bimonthly in juvenile horses [12]. Because macrocyclic lactones are larvicidal against *Parascaris*, the *refugia *within a host is minimized each time an infected foal is dosed. This happens routinely whenever the interval between treatments is shorter than the prepatent period for *Parascaris *(i.e., 75-80 days). In addition, susceptible genotypes in the local population are denied an opportunity to reproduce whenever macrocyclic lactone treatments are repeated at intervals which are less than the prepatent period or egg reappearance period, thus minimizing *refugia *in the environment. Typical parasite control practices for juvenile horses at many breeding operations essentially constitute exclusive and/or excessively frequent use of a single drug class, and thus select intensively for anthelmintic resistance [4].

## Transmission among facilities

It is likely that macrocyclic lactone resistance arose independently at multiple locations, and may do so again at any facility where traditional control practices are followed. As the prevalence of macrocyclic lactone-resistant ascarids increases, farms are at ever greater risk of inadvertently importing a resistant isolate.

The major potential source is foals which harbor immature infections. Fecal examination of such animals would be fruitless because their worm burdens are not yet capable of sexual reproduction. This particular route of dissemination is a great threat to the Thoroughbred industry, which requires that offspring must be sired by natural service in order to be registered. This requirement results in significant traffic of mares, with foals-at-side, to breeding facilities for natural service by a stallion. If a foal acquires a macrocycliclactone resistant ascarid infection at the breeding farm, it will transport it back home, and only time will reveal its presence. Treatment of returning foals with ivermectin or moxidectin is ineffective because the target infection is ML-R. Carefully timed administration of non-ML anthelmintics could reduce the number of resulting adult worms, but probably would not eliminate them totally.

## Detection of resistant isolates

Fecal flotation is a simple, inexpensive, and widely available procedure for detecting patent *Parascaris *infections in horses. Quantitative procedures (e.g., McMaster, Modified Stoll, Sucrose Centrifugation) provide valuable information regarding the magnitude of environmental contamination by individual animals. However, a correlation between egg counts (eggs per gram; EPG) and worm burdens has not been demonstrated for *P. equorum*, so one may not assume that horses with high egg counts are harboring large numbers of mature ascarids.

The Fecal Egg Count Reduction Test (FECRT) is the standard method for detecting anthelmintic resistance in cyathostomin nematodes of horses, but this procedure has not been validated for *Parascaris*. Nevertheless, FECRT is the only currently available test for quantifying anthelmintic removal of reproducing, adult, female *Parascaris *from an individual horse.

*Parascaris *FECRT can only be performed with horses that have positive egg counts, and some minimum quantitative standard (e.g., ≥ 200 EPG) should be established for inclusion in FECR calculations. Enrollment of large numbers of horses in an efficacy evaluation will provide a more accurate representation of the susceptibility status of the resident ascarid population. Following determination of pretreatment fecal egg counts, each candidate is treated according to label directions with the anthelmintic to be screened. Between 14-21 days after treatment, fecal samples are collected from the same individuals that were screened pretreatment, and fecal egg counts are repeated. Fecal Egg Count Reduction (FECR) is a measure of anthelmintic efficacy, expressed in percentages, and is calculated by the formula:



The magnitude of egg count reduction which comprises acceptable efficacy is generally accepted as >90% or >95% FECR. These ranges constitute rough guidelines only, but will have to serve until FECRT has been validated for *Parascaris*.

Anthelmintic resistance appears to be a permanent genetic feature of a parasite population, and reversion to susceptibility may never occur. Accordingly, if the resident ascarid population is resistant to a particular drug class, products from that chemical group should never again be used alone for ascarid control on those premises. However, drugs to which ascarids are resistant may retain substantial efficacy against other important equine parasites, such as large strongyles or cyathostomins. Any drug classes that are known to be effective against the indigenous ascarid isolate should be evaluated annually for continued efficacy.

## Control recommendations

Ideally, a decision to administer anthelmintics for removal of *P. equorum *infections would be based on a positive diagnostic result (e.g., fecal examination) for each animal to be treated. However, confirmation of patency also indicates that the environment is being contaminated with highly persistent ascarid eggs, which confounds the universal objective of parasite control. Ultimately, compromise is unavoidable, and some level of contamination must be accepted because suppressive programs select too intensively for the development of resistance. And, whenever treatment is indicated, it is desirable to use only anthelmintics with known efficacy against indigenous parasite populations.

The specter of *Strongyloides westeri *infection is not sufficient justification for deworming foals with MLs during the first month of life. *Strongyloides *is relatively uncommon and only occasionally pathogenic. Initial treatment of foals for *Parascaris *infection should not begin earlier than 60 to 70 days of age, and treatments thereafter should be repeated at the longest intervals which minimize environmental contamination with ascarid eggs.

One important feature of ascarid biology that should be considered in scheduling *Parascaris *treatments is that anthelmintic efficacy apparently increases as the target population ages. For example, oxibendazole (10 mg/kg) was 94% [13] to 100% [22] effective against patent (i.e., mature) ascarid infections when measured by FECRT. However, the same dosage removed only 44.5% of immature ascarids when administered at 28 days post-infection [23]. So, it is logical that anthelmintic treatments would be more effective against ascarids if administered just prior to patency, i.e., at 70 to 75 days post-infection. This knowledge has limited practical application, however, because natural infections "trickle" into the host, with multiple exposures occurring continuously on a daily basis. A foal with a negative fecal result could harbor ascarid populations ranging in age from 1 to 75 days, and anthelmintics directed against such a mixed population would likely remove the older ascarids but demonstrate little efficacy against juvenile worms.

Traditional recommendations for ascarid control are to treat foals at bimonthly intervals (i.e., q ~60 days), but this schedule may be insufficiently frequent to minimize the passage of eggs in the feces of some foals. However, deworming more frequently, especially with macrocyclic lactone anthelmintics, minimizes *refugia *and selects for resistance. It may be preferable to tolerate some level of egg contamination, because a survey in the Netherlands found little ML resistance on farms where foals were de-wormed less frequently than at bimonthly intervals [24].

If anthelmintic resistance is not an issue, acceptable efficacy can usually be achieved with any of the products listed in Table 1.

**Table 1:** Chemical class, generic name, and dosage of anthelmintics with label claims for efficacy against *Parascaris equorum*.

**Chemical class**	**Generic name**	**Dosage**
Benzimidazoles	Fenbendazole	10 mg/kg
	Oxibendazole	10 mg/kg
Pyrimidines	Pyrantel pamoate	6.6 mg/kg
	Pyrantel tartrate	2.64 mg/kg/day
Macrocyclic Lactones	Ivermectin	200 μg/kg
	Moxidectin	400 μg/kg
Heterocyclic Compounds	Piperazine	88 mg/kg

If ML-R ascarids are present on a farm, benzimidazole or pyrimidine formulations can be administered easily and usually provide good efficacy. Rotation between effective drug classes is recommended [25,26]. Recently, ML-R *Parascaris *populations that are simultaneously resistant to pyrantel pamoate have been reported from Texas [12] and Kentucky [13]. For these populations, the only remaining, effective drugs are piperazine, fenbendazole, or oxibendazole. Due to the possibility of multiple drug resistance, the continuing efficacy of all drug classes used against *Parascaris *should be confirmed annually on each farm.

Preventing the introduction of a ML-R strain to a farm is particularly difficult to manage, because the infection cannot be detected and efficacy cannot be verified. Furthermore, non-ML anthelmintics have no efficacy against migrating stages during the first month post-treatment, and only partial efficacy thereafter until the population becomes fully mature.

A regimen of fenbendazole, 10 mg/kg daily for five consecutive days represents one possible tool for preventing the inadvertent introduction of a resistant isolate. A previous study demonstrated that this regimen of fenbendazole was 99.7% effective when administered between 11-15 days post-infection [27]. Although multiple-day fenbendazole is not specifically approved for removal of immature *Parascaris *infections, it is labeled for larvicidal therapy of migrating large strongyles and encysted cyathostomins. The suggested prophylactic uses of this regimen include treatment of foals when they return with their dams from a breeding facility, or treatment of any juveniles upon first introduction to a new facility.

## Possible biological changes

Anthelmintic resistance is only one manifestation of genetic change in a parasite population in response to various selection pressures. Other biological adaptations are certainly feasible, and some could even impact practical control more deleteriously than drug resistance. For instance, acquired immunity is the ultimate ally in controlling equine ascarids, but if *P. equorum *isolates with low immunogenicity were to evolve, the challenges of ascarid control could extend to horses of all ages, rather than just juveniles. Variations from the typical host age spectrum have been reported with *Oxyuris equi*, and altered immunity is one feasible explanation [28].

It is also possible that the prepatent period or egg reappearance period of *Parascaris *could become abbreviated as a response to frequent anthelmintic treatment. This phenomenon has not yet been investigated in ascarids, but reduction of the egg reappearance period of cyathostomins has been documented as a consequence of anthelmintic selection pressure [8,15,29-32].

The present and emerging threats associated with anthelmintic treatment lend particular urgency to the development of sustainable approaches to parasite management which are not exclusively dependent on chemical control.

## Conclusion

The development of anthelmintic resistance in some populations of *P. equorum *means that casual selection of dewormers must be discontinued, and that treatments can no longer be administered at frequent intervals. In the future, the resistance status of each drug class should be evaluated against local isolates, and efficacy should be re-confirmed at regular intervals. The Fecal Egg Count Reduction Test is a simple procedure which can be adapted for this purpose. Although fecal monitoring will increase the costs of administering control programs, the alternative, i.e., expanding resistance, is unacceptable. Future management of the entire spectrum of equine parasites lies in the development of sustainable approaches which do not rely solely on anthelmintic treatment.

## Competing interests

Fort Dodge Animal Health financed the article-processing charges for this paper. The author declares that he has no other competing interests.

## Author's information

CRR is President of a veterinary Contract Research Organization which conducts development research to support the regulatory approval of parasiticides and other pharmaceuticals for domestic animals.
